# Mental Health Literacy in Zurich: A First Measurement Attempt Using the General HLS-EU-Q47

**DOI:** 10.3389/fpubh.2021.723900

**Published:** 2021-09-13

**Authors:** Michael Schneider, Rebecca Jaks, Daniela Nowak-Flück, Dunja Nicca, Saskia Maria De Gani

**Affiliations:** ^1^Department of Health Sciences and Technology, Institute of Human Movement Sciences and Sport, Eidgenössische Technische Hochschule Zurich, Zurich, Switzerland; ^2^Health Literacy Division, Careum Foundation, Zurich, Switzerland; ^3^Epidemiology, Biostatistics and Prevention Institute, University of Zurich, Zurich, Switzerland

**Keywords:** mental health literacy, health literacy, measurement tool, health behavior, health outcome

## Abstract

**Background:** Mental health literacy (MHL) promises to be an important factor for public health by enabling people to take responsibility for their own mental health. To date, there is no measurement tool that allows the assessment of a comprehensive understanding of MHL as part of health literacy (HL). Nonetheless, the widely used Health Literacy Survey European Questionnaire 47 (HLS-EU-Q47) includes items assessing at least some MHL-aspects in the context of HL. The present study aimed at investigating how these MHL-aspects are related to HL, health behavior and health outcome and how they differ between sociodemographic groups.

**Methods:** Data from the Health Literacy Survey Zurich 2018, collected by an adapted version of the HLS-EU-Q47, served to investigate these relationships.

**Results:** MHL-aspects were related to HL, health behavior and health outcome. Nearly half of all respondents (45%; *N* = 904) showed low MHL levels, particularly those with higher age and higher financial deprivation.

**Conclusions:** Relations of MHL-aspects with HL, health behavior, and health outcome indicate their potential importance for future interventions in public health, addressing mental health and MHL. A specific MHL tool is needed to comprehensively investigate these relations, which could be developed by extending the present measurement approach.

## Introduction

Mental health is an essential requirement for good health. Therefore, it is an integral vision of the World Health Organization (WHO) to achieve the highest possible standard of mental health and well-being for the entire population (1). Nowadays, mental health conditions cause one fifth of all years lived with disability worldwide (1), and have a significant impact on the quality of life of the affected individuals and their families (2). In Switzerland, 15% of the population report moderate to severe mental stress, while around three quarters of those with severe mental stress or depressive symptoms suffer from physical complaints as well (3). Considering that respondents of the Swiss Corona Stress Study (4) reported an increase of stress and depressive symptoms during the COVID-19 pandemic, the psychological burden of the Swiss population may even be higher.

Mental health literacy (MHL) promises to be an important resource to cope with this burden, as it may not only facilitate recognition of mental disorders and early help-seeking (5), but possibly also promote mental health (6).

MHL can be considered as an integral part of health literacy (HL), which itself can be understood as an individual's motivation, knowledge and ability to find, understand, and use health information to manage one's own health through informed decisions and corresponding health behavior (7). Hence, HL focuses on competencies in dealing with health information and exceeds aspects only linked to disease management. In fact, HL also includes the two dimensions disease prevention and health promotion that are important for both mental and general health. As the concept of HL is still discussed diversely (7, 8), also MHL has been explored with different definitions so far (9, 10). A common definition states that MHL includes the ability to recognize specific mental disorders, knowledge of risk factors, causes, self-treatments, availability of professional help, knowledge on how to seek mental health information as well as attitudes promoting recognition and appropriate help-seeking (11). Additionally, there have been discussions on an extended definition of MHL that does not only include knowledge and beliefs about mental disorders (12–14). Accordingly, Kutcher et al. (13) defined four main components of MHL: (1) understanding how to obtain and maintain positive mental health, (2) understanding mental disorders and their treatments, (3) decreasing stigma related to mental disorders, and (4) enhancing help-seeking efficacy, which means knowing when and where to seek help and developing competencies designed to improve one's mental healthcare and self-management capabilities. Including the understanding of how to obtain and maintain a good mental health in the definition of MHL is in line with the comprehensive concept of HL as well as the WHO's definition of mental health (15), i.e., mental health is more than the absence of mental health disorders.

The identification of specific sociodemographic groups reporting low MHL levels is important for the initiation of targeted interventions to strengthen their abilities to care for their own mental health. To identify whether people with a low level of MHL might also report needs concerning general health, it is also important to investigate their health behavior and health outcome. While many studies examined the relationship of HL and sociodemographic factors (16, 17), health behavior (16–19), and health outcome (16, 17, 20), so far only few studies examined these aspects in relation to MHL. These studies related MHL-aspects to sociodemographic characteristics such as age (21–23), gender (21–24), education (21–23), financial situation (23, 25), and rural residence (24, 26). Studies investigating MHL-aspects in the context of health behavior showed that stigma could be associated with more frequent alcohol and drug abuse (27), and low rates of help-seeking could be associated with higher rates of substance use disorders (28). In contrast, the few studies on MHL and health outcome showed that on the one hand higher MHL levels were related to better (self-assessed) health (23, 29), and on the other hand, inadequate MHL levels were associated with increased odds for moderate to severe depression (30). In summary, so far studies on MHL used only few measures of health behavior and health outcome and merely focused on specific subpopulations. Additionally, these studies were based on different definitions of MHL and mostly omitted the aspect of positive mental health.

In addition, these studies investigated MHL with different measurement tools (10). However, to date, no specific instrument can be found which assesses the comprehensive spectrum of MHL as part of general HL. Moreover, most studies so far have related MHL only to specific sociodemographic characteristics or few aspects of health behavior and health outcome. Nonetheless, the widely used instrument to assess general HL—the so-called Health Literacy Survey European Questionnaire 47 (HLS-EU-Q47) (31)—includes at least some MHL-aspects in the context of HL. The HLS-EU-Q47 is usually applied to assess general HL including its specific abilities to access, understand, appraise and apply health information across the areas of healthcare, disease prevention and health promotion (16). Containing only few items that consider aspects of MHL, the questionnaire originally was not constructed to holistically assess MHL. However, it offers the opportunity to assess some MHL-aspects and their relation to HL, several sociodemographic characteristics and aspects of health behavior and health outcome. Therefore, the aim of the present study was to make a first attempt to examine MHL in the population of Zurich using this instrument and the MHL-aspects as well as their relation to general HL, health behavior, health outcome, and sociodemographic characteristics. For this purpose, recent data from a study on general HL of the population of the canton of Zurich—“Health Literacy Survey Zurich” (HLS-ZH-18) (32)—was used.

## Materials and Methods

### Study Population

For the analysis, data of the population survey HLS-ZH-18 was used. Parts of the data have been analyzed and published in another context (32), other data has remained unpublished so far. The study population consisted of a total of 1,000 residents of the canton of Zurich (Switzerland) aged 18 years or older. Participants were interviewed between November and December 2018 using Computer Assisted Personal Interviews (CAPI) in German language. Data was collected by a third party (gfs.bern AG, research institute, Bern, Switzerland), which had also collected the data for the “Swiss Health Literacy Survey” (HLS-CH-15) (17). The present sample size was considered to be enough in order to conduct population- and subgroup analyses. Sampling error was 3.2. Sampling was conducted by a random selection of 100 cantonal sampling points and predefined quotas on site (age, gender). Communities with at least 1,000 residents built the basis for the sampling points. Larger communities had several sampling points (one for every 1,000 residents). The type of settlement was also taken into account when drawing the sampling points. A total of ten interviews per sampling point were conducted. Trained interviewers randomly interviewed pedestrians, whereby interviewers were free to choose where they contacted the participants. The mean duration of the interview was 30.3 (± 6) min. Participants were verbally informed about the goals, framework conditions and data protection measures before they gave their informed consent to participate in this study. All processes were in line with the legal and association requirements for the protection of data and personal rights (VSMS). A separate ethical approval for this study was not necessary.

### Questionnaire

The HLS-ZH-18 questionnaire was based on the national survey HLS-CH-15 (17), which in turn consisted of the 47 adapted HL items of the HLS-EU-Q47 (31). All of these self-assessment-instruments served to assess HL as well as health behavior, health outcome and sociodemographic characteristics.

#### MHL-Associated Items

The HLS-EU-Q47 and the HLS-CH-15 questionnaire do not contain a specific MHL module so far. Therefore, in the present study (HLS-ZH-2018), four items related to mental health or MHL, respectively, could be identified and are referred to as “MHL-associated items.” These four items (Q4, Q18, Q33, and Q40) built the focus of the present study (Table 1).

**Table 1:** MHL-associated items from the HLS-ZH-2018.

**Item**	**On a scale from very easy to very difficult, how easy would you say it is to:**
Q4^*^	“…*find out where to get professional help when you are ill? (doctor, pharmacist, and psychologist)*”
Q18	“…*find information on how to manage mental health problems like stress or depression?*”
Q33	“…*find out about activities that are good for your mental well-being? (meditation, exercise, walking, Pilates etc.)*”
Q40	“…* how easy would you say it is to understand information on how to keep your mind healthy?*”

* *Although Q4 can be related also to MHL, participants' answers to it may refer not only to help from a psychologist but also from a doctor or a pharmacist. This has to be considered when interpreting the data*.

#### MHL-Index, General HL-Index, and HL-Index

Out of the four MHL-associated items Q4, Q18, Q33, and Q40 an MHL-Index was built. As a second index, the general HL-Index (HL_47_) including all 47 HL items was built. The third index that was built was the HL-Index (HL_43_) and included 43 HL items, without the four MHL-associated items. All items were assessed with a Likert scale and numerical values were accordingly assigned (“very easy” = 4, “fairly easy” = 3, “fairly difficult” = 2, “very difficult” = 1). Based on these values, corresponding indices for each individual were built by calculating the mean and then applying the following formula, as recommended by the HLS-EU consortium (16, 33):


$$\begin{array}{l} Index= \end{array}(\text{mean}-1)\times \frac{50}{3}$$

Accordingly, the indices were only calculated if a minimum respondent rate of 80% in all 47 HL items was achieved and all four MHL-associated items were rated as well. These criteria resulted in the inclusion of 904 participants. In a novel approach, the here calculated MHL-Index was interpreted like the standard general HL-Index (HL_47_), which means that 0–25 points were rated as “inadequate,” >25–33 as “problematic,” >33–42 points as “sufficient” and >42–50 points as “excellent” MHL or HL, respectively (16, 33).

For the multiple logistic regression analysis, the MHL-Index was also defined dichotomously, whereby the categories “excellent” and “sufficient” (>33–50 points) were summarized as “high MHL” and “problematic” and “inadequate” (0–33 points) were summarized as “low MHL”.

Cronbach's alpha for all 47 items was 0.889, indicating a high level of internal consistency. For MHL-associated items Cronbach's alpha was 0.547.

#### Sociodemographic Characteristics, Health Behavior, and Health Outcome

Sociodemographic characteristics as well as health behavior and health outcome were assessed with the same questions (except for minor changes) and scales as in the HLS-CH-15 (17). Included sociodemographic variables were age, gender, education, financial deprivation, and type of settlement. Included health behavior variables were smoking behavior, alcohol consumption and physical exercise frequency. Body-mass-index (BMI), self-assessed health status and presence of chronic disease were included as health outcome variables.

For the multiple logistic regression analysis, several variables had to be re-categorized: (1) educational levels that have been classified according to the International Standard Classification of Education (ISCED) (34) were divided into the three categories low (level 0–2), medium (level 3–4) and high (level 5–6) education; (2) alcohol consumption was categorized as excessive (very excessive, excessive) or non-excessive (moderate, low, no alcohol); (3) physical exercise frequency was categorized into weekly (each day to a few times a week) and less than weekly (a few times per month to not at all); and (4) self-assessed health status was categorized as bad (very bad, bad), medium or good (good, very good).

### Data Analysis and Statistics

Collected data were weighted according to the sociodemographic characteristics age/sex interlocked, type of settlement and highest level of education to account for the sample design, to adjust for respective sociodemographic characteristics and to increase representativity of the results. The Federal Statistical Office's statistics served as a reference for the weights (35, 36). Descriptive statistical analysis was used to characterize the sample, to analyze answer frequencies regarding MHL-associated items and to investigate MHL levels of the study population and their associations with HL_47_. Subgroup analysis with <50 respondents was—whenever possible—avoided. The interpretation of this analysis was almost impossible because of sampling errors of ±14 percentage points. Hence, when smaller subgroups were identified, this was explicitly pointed out.

To investigate associations of MHL with HL_47_, HL_43_, and single HL items, spearman's rank correlation coefficients were calculated. In this context, HL_43_ was used to investigate the association of MHL and HL—thus HL independent of the four MHL-associated items. To evaluate the significance and directions of the associations between MHL and health behavior, health outcome and sociodemographic characteristics, spearman's rank correlation coefficients were calculated. For all spearman's rank correlation coefficients, respective variable scales were defined in ascending order. In a second step, associations of MHL (dependent variable) with health behavior, health outcome and sociodemographic characteristics (independent variables) were assessed in a multiple logistic regression model. This step allowed the comparison of the odds ratio (OR) of different subgroups for having low MHL levels. Corresponding listwise exclusion led to a sample of 831 respondents in total. Assumptions for all conducted statistical tests were fulfilled. The response category “do not know” was interpreted as a missing value. Respective tests were two-sided and for multiple logistic regression 95% confidence intervals (CI) for OR were calculated.

Statistical analysis was conducted with the IBM SPSS v.26 software (IBM Corp. Armonk, NY, USA). For all statistical analyses, a value of *p* < 0.05 was considered significant. Results are presented as mean ± standard deviation (SD), percentage (%), spearman's rank correlation coefficient (*r*_*s*_), *p*-value, OR, and CI.

## Results

### Characteristics of the Study Group

Overall, 904 participants were included in the analysis. Study participants were 46.2 ± 18.0 years old. The youngest respondent was 18 and the oldest 88 years old. Sociodemographic characteristics of the included study population are presented in Table 2.

**Table 2:** Sociodemographic characteristics of the included study population.

	**Total (*N* = 904)**
	**% (*n*)**
**Gender**	
Female	51% (456)
Male	49% (448)
**Age**	
18–39	37% (341)
40–64	42% (386)
65+	21% (177)
**Education**	
Low	20% (66)
Medium	43% (662)
High	37% (176)
**Type of settlement**	
Rural	5% (47)
Small/mid-sized city	16% (156)
Big city	80% (701)

*(n), unweighted number of cases; %, weighted percentage of a total of 904 respondents*.

*Percentages are rounded mathematically and do not always add up to exactly 100%*.

### MHL of the Population of Zurich

**Figure 1:**
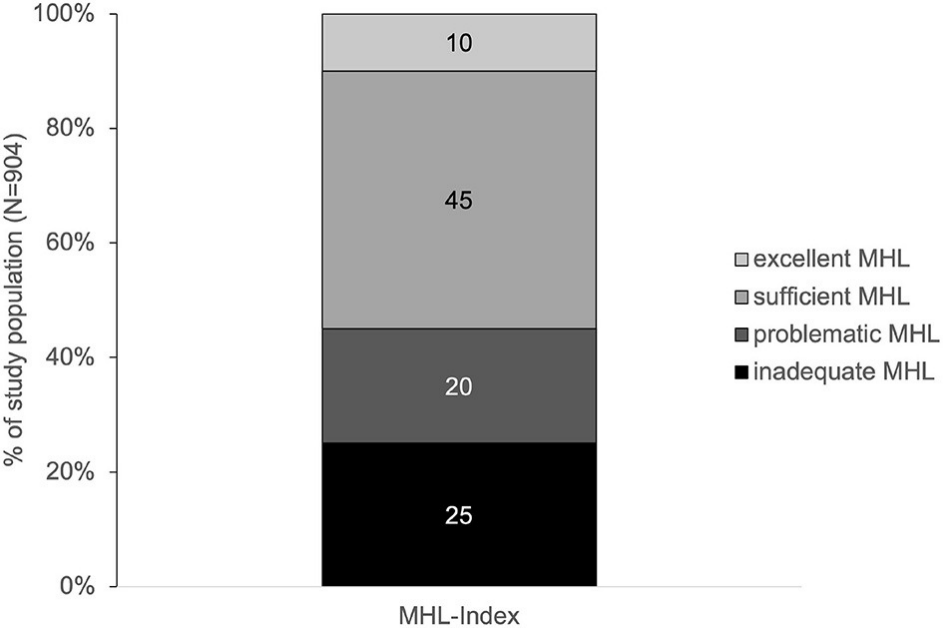
MHL levels in percentage of the study population.

The average MHL of men and women in Zurich was inadequate (32.6 ± 8.3). Accordingly, nearly half of all respondents showed a problematic or inadequate MHL (Figure 1).

**Figure 2:**
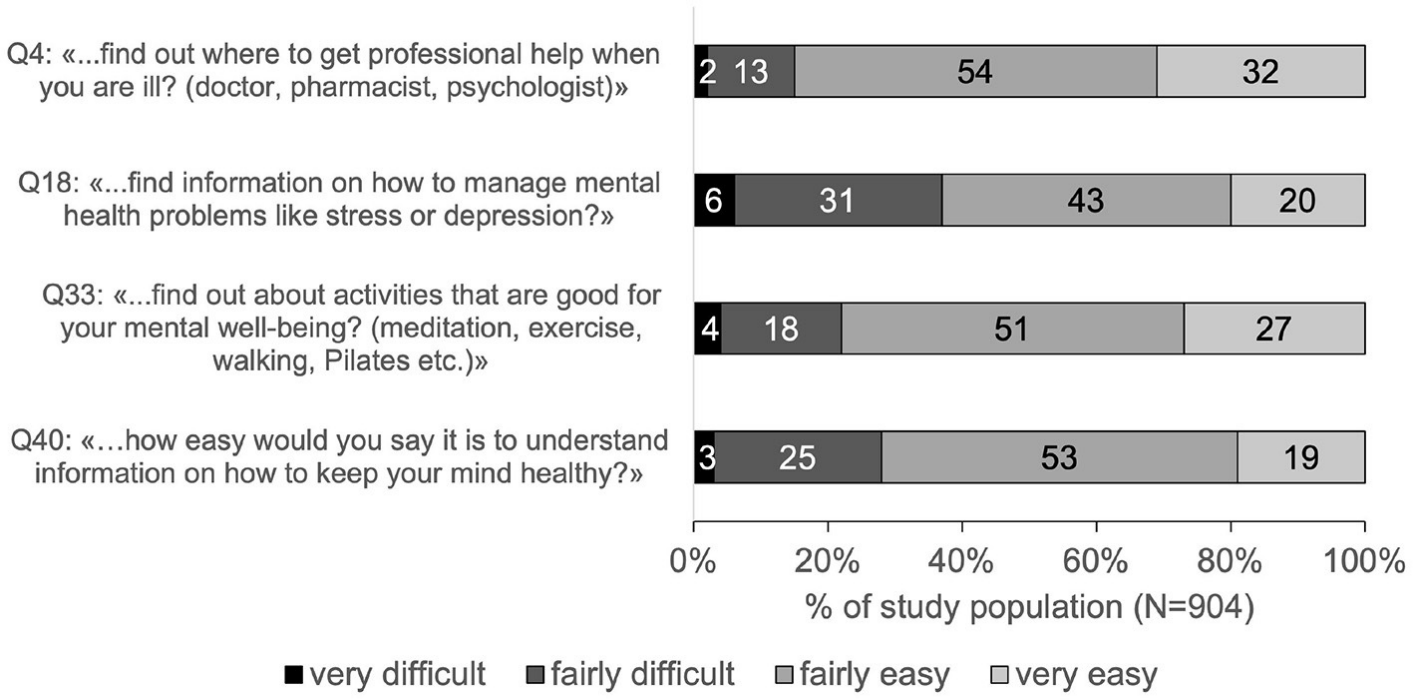
Ratings of MHL-associated items in percentage of the study population. Percentages are rounded mathematically and do not always add up to exactly 100%.

Most difficulties (37%) were reported with “…*find information on how to manage mental health problems like stress or depression?*” (Q18, Figure 2). Least difficulties (15%) were reported with “…*find out where to get professional help when you are ill? (doctor, pharmacist, and psychologist)*” (Q4, Figure 2).

### MHL and HL

**Figure 3:**
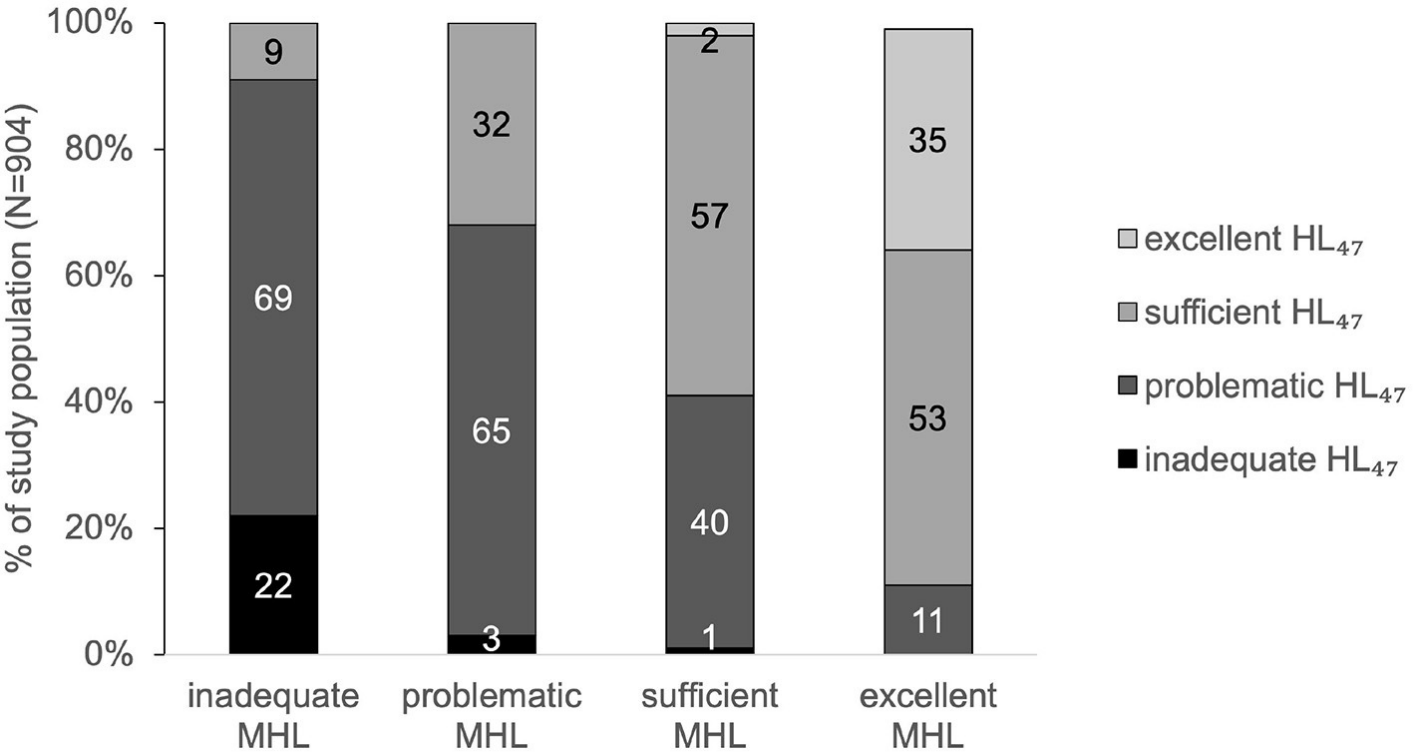
Association of MHL and HL_47_.

Ninety-one percent of the participants with inadequate MHL showed inadequate or problematic HL_47_. In contrast, 88% of the participants with excellent MHL showed sufficient or excellent HL_47_ (Figure 3).

MHL significantly correlated to HL_43_ (*r*_*s*_ = 0.563, *p* < 0.001). In addition, MHL showed the strongest correlations with the HL_43_ items Q2, Q17, Q20, and Q32 (Table 3).

**Table 3:** Analysis of the strongest correlations between MHL and single items of the HL_47_.

**Item**	**Item description**	***r*_*s*_** ^*^	***p*-value**
Q2	“… *find information on treatments of illnesses that concern you?*”	0.387	<0.001
Q17	“… *find information about how to manage unhealthy behavior such as smoking, low physical activity, and drinking too much?*”	0.377	<0.001
Q20	“… *find information on how to prevent or manage conditions like being overweight, high blood pressure, or high cholesterol?*”	0.400	<0.001
Q32	“… *find information on healthy activities such as exercise, healthy food and nutrition?*”	0.404	<0.001

* *Spearman's rank correlation coefficient (r_s_)*.

### MHL and Its Relations to Sociodemographic Characteristics, Health Behavior, and Health Outcome

Compared to general HL, MHL showed correlations with the same direction but lower strength with all sociodemographic characteristics, except for rural residence. Hence, MHL was positively correlated to education (*r*_*s*_ = 0.167, *p* < 0.001), and negatively correlated to age (*r*_*s*_ = −0.173, *p* < 0.001) as well as financial deprivation (*r*_*s*_ = −0.307, *p* < 0.001). No association was found between MHL and type of settlement (*r*_*s*_ = 0.012, *p* = 0.688). Significant effect sizes were found for age and financial deprivation (Table 4): Participants aged 65 years and older (OR = 2.542, 95% CI: 1.509–4.282) and those with high financial deprivation (OR = 2.314, 95% CI: 1.560–3.432) were more than twice as likely to have low MHL than younger residents and participants with low financial deprivation.

**Table 4:** Associations of MHL and sociodemographic characteristics, health behavior, and health outcome.

**Independent variable**	**Proportion** ^**a**^ **% (*n*)**	**OR**	**95% CI**		***p* value** ^**b**^
			**Lower**	**Upper**	
**Gender**					
Female^*^	50% (414)	1	–	–	–
Male	50% (417)	0.939	0.677	1.303	0.709
**Age**					
18–39^*^	37% (307)	1	–	–	–
40–64	44% (368)	0.935	0.650	1.344	0.715
65+	19% (156)	2.542	1.509	4.282	0.000
**Education**					
Low^*^	7% (55)	1	–	–	–
Medium	73% (607)	1.353	0.853	2.144	0.199
High	20% (169)	1.636	0.999	2.679	0.050
**Financial deprivation**					
Low^*^	32% (268)	1	–	–	–
Middle	21% (173)	1.584	1.001	2.506	0.049
High	47% (390)	2.314	1.560	3.432	0.000
**Type of settlement**					
Big^*^	78% (647)	1	–	–	–
Small/medium-sized	17% (142)	0.446	0.287	0.695	0.000
Rural^c^	5% (42)	1.152	0.554	2.393	0.705
**Smoking behavior**					
Non-Smoker^*^	25% (205)	1	–	–	–
Ex-Smoker	23% (190)	0.956	0.598	1.527	0.850
Smoker	52% (436)	1.547	1.040	2.301	0.031
**Alcohol consumption**					
Non-excessive^*^	87% (720)	1	–	–	–
Excessive	13% (111)	0.643	0.400	1.031	0.067
**Physical exercise frequency**					
Weekly^*^	33% (274)	1	–	–	–
Less than weekly	67% (557)	2.214	1.532	3.200	0.000
**BMI**					
Normal weight^*^	65% (544)	1	–	–	–
Overweight	29% (242)	1.856	1.291	2.668	0.001
Obesity^c^	5% (45)	2.014	1.060	3.826	0.032
**Health status**					
Good^*^	77% (643)	1	–	–	–
Medium	18% (147)	1.115	0.652	1.905	0.691
Bad^c^	5% (41)	0.753	0.322	1.760	0.512
**Chronic disease**					
No^*^	75% (621)	1	–	–	–
Yes	25% (210)	0.790	0.476	1.310	0.360

a *(n), number of cases per subgroup; %, percentage of a total of 831 included respondents*.

b *p-value for multiple logistic regression analysis with MHL as dependent variable [low vs. high MHL (=reference category)]*.

c *Subgroup analysis with less than 50 respondents*.

* *Reference categories for odds ratio analysis*.

*Sociodemographic characteristics are colored white, health behavior with light gray and health outcome with dark gray*.

*Percentages are rounded mathematically and do not always add up to exactly 100%*.

In addition, and again compared to general HL, in the most cases MHL showed correlations of the same direction with health behavior and health outcome. Hence, MHL was positively correlated to physical exercise frequency (*r*_*s*_ = 0.254, *p* < 0.001) and self-assessed health status (*r*_*s*_ = 0.263, *p* < 0.001). In contrast, smoking behavior (*r*_*s*_ = −0.130, *p* < 0.001), BMI (*r*_*s*_ = −0.066, *p* < 0.05), and occurrence of chronic disease (*r*_*s*_ = −0.161, *p* < 0.001) were negatively correlated with MHL. No association was found between MHL and alcohol consumption (*r*_*s*_ = 0.003, *p* = 0.910). Significant effect sizes were found for physical exercise frequency, smoking behavior, and BMI (Table 4). Individuals who reported to be physically active less than once per week were more likely to have low MHL than their counterparts (OR = 2.214, 95% CI: 1.532–3.200). Smokers and overweight individuals were more likely to have low MHL compared to non-smokers and individuals with normal weight.

## Discussion

Nearly half of the study population reported low MHL: 20% showed problematic and 25% inadequate MHL. Thus, a substantial part of Zurich's population seems to have considerable difficulties with handling information on mental health. The main difficulty hereby concerned the access to information on how to cope with mental health problems. A similar result could be found in the Swiss national study HLS-CH-15 (17). Concerning MHL, also the Swiss population reported most difficulties in finding information on how to manage mental health problems like stress or depression (27% in HLS-CH-15 vs. 37% in HLS-ZH-18). Considering these results, it seems crucial for the entire population to facilitate access to information on mental health. Knowing where to find information on coping strategies is a first step toward learning and applying such strategies to deal with mental health issues. This is even more important in respect of the increasing number of people with mental health problems (4) and other factors that could hinder help-seeking, like stigma for example (37) that seems to be still high in Switzerland (38). Furthermore, a lack of knowledge on strategies to deal with mental health problems has recently been detected in the younger Swiss population (39). This lack of knowledge could possibly also count for the general population. Thus, strengthening the access to information related to this knowledge seems to be of great necessity.

The present study population also showed difficulties in accessing and understanding information on the promotion and maintenance of their mental health. More than a fifth (22%) reported difficulties with finding information about activities that are good for their mental wellbeing (Q33) and more than a quarter (28%) reported difficulties with understanding information on how to keep their own mind healthy (Q40). Considering that during a pandemic like the COVID-19 pandemic, possibilities of mental health promoting activities, as for example meeting friends, or participating at community sports activities, might be restricted, it can be expected that finding appropriate mental health promoting activities might even be more difficult. In this context another recent Swiss survey concluded that there may not only be a lack of factual knowledge, but also concrete knowledge for action for mental health promotion (38). For example, only 46% of the respondents reported that they knew how to strengthen their mental health. Therefore, it seems to be important to not only offer alternative mental health promoting activities but also to make people aware of them, facilitate access to them, and increase the understanding of their importance. The current study as well as the Swiss national study on general HL showed that people report more difficulties with appraising and applying health information rather than with finding and understanding them. Connecting this to the present findings, one might expect that if the assessment of MHL would have also included the two domains of appraising and applying information on mental health, MHL levels of the population might have even been lower and more problematic.

The results of this study indicate that MHL can be associated with general HL. Most of the respondents with inadequate MHL also showed inadequate or problematic general HL. This correlation was found to be true, irrespective of whether the four MHL-associated items were included into the model of HL (HL_47_) or not (HL_43_). This indicates that people with low MHL often not only seem to have difficulties with finding and understanding information on mental health, but also with handling health information in general. People with low MHL therefore possibly may need to be supported not only in their abilities to care for their mental health but in a more comprehensive manner, including their physical health. Furthermore, the relation between MHL and HL seems to support the understanding of MHL as an integral part of HL. This relationship between MHL and HL needs to be carefully treated, however, as the questionnaire did mainly focus on general HL and did not include a comprehensive conceptualization of MHL, but a rather limited number of MHL-associated items. Nonetheless, the present findings are in accordance with another study that also showed a substantial association between MHL and HL (22). In addition to the present approach, the referred study considered HL as a predictor of MHL. The authors pointed out that poor HL could be associated with greater prevalence of mental illness symptoms and a lower likelihood to seek professional help for these symptoms. In the present study, however, HL is not understood as an antecedent for MHL or vice versa, as for example the ability to handle information on general health does not necessarily influence the ability to handle information on mental health. It is rather hypothesized that personal, situational, societal, and environmental determinants that have an influence on HL (7), may also determine MHL.

The present study found older age, lower education, and higher financial deprivation to be associated with low MHL and low HL. Low MHL in older and lower educated in Switzerland were also found in another survey which stated that they report more pronounced difficulties in understanding information on mental disorders (38). Reasons for low HL in these subgroups may at least also partly be responsible for low MHL. In other words, the pronounced difficulties with higher age regarding dealing with general health information as well as accessing and understanding information on mental health may be explained by an age-dependent decline of cognitive abilities (40). Furthermore, health information is increasingly often available online. Accessing this information and assessing the quality of online health information seems to be a great challenge, especially for the elderly (41). Another factor that might affect the access and understanding of information on mental health in general, but especially at higher age, is stigma. Actually, stigma has been seen as a significant barrier to access care in case of mental disorders in elderly people (42), whereby especially Swiss people over 80 years seem to be affected by stigmatization (38). In this context, the WHO, the World Psychiatric Association and the Swiss Society for Public Health have emphasized the importance of destigmatization (42, 43). Assuming that stigmatization may have decreased (44), destigmatization is still ongoing, and awareness of mental health issues is rising, MHL could possibly profit thereof in the future. Apart from the present results, weak depressive symptoms (45) as well as medium to high mental stress seem to increase with the years after retirement (46). This further indicates the great need to strengthen MHL levels of the elderly. MHL of this population group could be strengthened by further decreasing stigma, increasing awareness of mental health issues as well as by facilitating and empowering them to access trustful and easy comprehensible (online) information on mental health.

Lack of awareness of mental health issues combined with stigma is also indicated in lower educated people (47–51), and may provide a possible explanation for their difficulties in accessing and understanding information on mental health. Respective subgroups showed higher levels of stigma (47), less knowledge (48), and poorer recognition of mental disorders (49–51). The need to improve MHL of low educated people seems to be especially important as they are more affected by mental stress (3, 52) and common mental disorders (53) than higher educated people. Besides low education, also high financial deprivation was associated with low MHL. This finding could be due to different reasons: Financially disadvantaged people may possibly less often use mental health services due to restricted access and financial reasons, and hence also be less aware of mental health issues. In addition, low financial capacities might also prevent them from participating in activities that could promote mental health.

Moreover, low HL was often found to be associated with poorer health behavior and outcome (16, 17, 20), whereas high HL was associated with more favorable health behavior and outcome (16–19). Therefore, it was hypothesized that low MHL (as part of HL) would be similarly associated with less favorable health behavior and worse health outcome. Accordingly, positive health behavior (reflected by higher physical exercise frequency and less smoking) and better health outcome (reflected by higher self-assessed health status, lower BMI, and fewer chronic diseases) were positively associated with MHL. One possible reason for the more unfavorable health behavior of individuals with low MHL could be their reported difficulty in finding information on changing an unhealthy or maintaining a healthy lifestyle (Q17, Q20, and Q32). Without or with less knowledge on health behavior and healthy lifestyle, it may be difficult and hardly possible to change one's behavior or maintain a healthy lifestyle. Nevertheless, knowledge on health behavior or on changing lifestyle does not automatically lead to healthier behavior. In this context, there are certainly other factors, for example motivational or situational factors that may influence MHL and health behavior. Moreover, less healthy lifestyle of participants with low MHL was also associated with poor health outcome, and worse health status as well as higher occurrence of chronic diseases were associated with lower MHL.

### Implications

Considering the present findings and the current substantial and increasing mental health burden, a measurement tool to assess and monitor MHL in a comprehensive approach is needed. The self-constructed MHL-Index of this study—based on the comprehensive model of HL—seems to be a promising first attempt. Additionally, although a correlation with general HL could be found, the present measure only included the domains finding and understanding of information on mental health issues. However, finding and understanding of such information without the ability of judging and applying it, is not sufficient to take responsibility for one's own mental and general health. In agreement with Mansfield et al. (10), it is therefore recommended that future MHL measures assess the ability to find, understand, appraise and to apply information on mental health to being able to take care of one's own mental health. Finally, the definition and assessment of MHL should comprise all relevant dimensions, including management of mental disorders, prevention thereof and also promotion of mental health. Thus, it could be recommended to extend the current HLS-questionnaire with an optional module including items capturing MHL across all these outlined domains and dimensions. The approach of incorporating current MHL constructs and definitions into a more holistic model may pave the way for a more unified research direction of MHL and HL in the future.

In addition, and in consideration of the limited MHL model used in this study, some first implications for public health in Zurich and Switzerland may be formulated as well. It could be revealed that almost half of the respondents showed low MHL levels. These levels might even be lower considering the missing assessment of the two domains appraising and applying of information on mental health. Moreover, the need to strengthen MHL in the general population might even be more important in respect to the increasing numbers of people with mental health problems, poor knowledge on management of mental health problems and difficulties in coping with this lack of knowledge. MHL could be strengthened by facilitating the access to information on mental health and to information which especially address topics like coping strategies for mental health problems and mental health promotion. In this context, destigmatization campaigns might play an important role in strengthening MHL as well. Anti-stigma interventions at the workplace for example have shown to be a promising approach by improving employees' knowledge and supportive behavior toward people with mental-health problems (54). Another promising option could be the initiation of tailor-made interventions like mental health promotion campaigns for specific population groups. It seems to be crucial to increase their MHL and HL in order to strengthen their ability to care for their own mental and general health at the same time. In this context for example, Health Promotion Switzerland (55) has highlighted the importance of tailored community health education events on mental health and offers easy comprehensible health information for different target groups. Further initiations are however needed.

### Limitations

There are certain methodological weaknesses that need to be considered when interpreting the present findings. First, due to the limited number of MHL-associated items, the present MHL-Index cannot be considered as a valid measure of a comprehensive MHL concept. Second, all data were self-reported and thereby carry the risk of reporting bias and social desirability. Additionally, quota and inclusion criteria may have only partially allowed for an unbiased selection of participants, as interviewers were free to choose the location of recruitment. Third, the use of German language only might have excluded people less competent in this language and might have led to a selection bias. Regarding data analysis, the transformation of ordinal and continuous into categorial data could have led to an exclusion of important information, despite the advantages of the multiple logistic regression as an adjusted analysis method. In addition, listwise exclusion led to reduced group sizes and might have affected the informative value, e.g., in the groups rural residence, poor self-rated health status or low education. Therefore, spearmen rank's correlation has been valued higher, as results were independent of categorizations and based on more individuals. Moreover, more information could be considered by including ordinal and continuous data. For future approaches and when statistical assumptions can be fulfilled, it might be recommended to rather make use of a linear regression method, or to make sure to include enough respondents for each category. Also, Cronbach's alpha of the MHL-associated items was quite low. This had to be expected because the items were not self-generated or composed to measure a predefined construct, nor did the index contain a great number of items. However, the single MHL-associated items were created in a logical, systematic and structured development process (31) and gave important insights into MHL of the population of the canton of Zurich. Finally, regarding the aspect of mental health, the present survey assessed rather unspecific information on health behavior and health outcome. Therefore, future MHL surveys should capture such variables more specifically, for example by asking for specific mental diseases, drug consumption, and addictions.

## Conclusions

The present study gives first insights into several aspects of MHL among residents of the canton of Zurich using an adapted version of the commonly used health literacy survey HLS-EU-Q47. A substantial number of individuals reported difficulties in handling information on mental health, which in turn was associated with lower HL, less favorable health behavior and poorer health outcome. Therefore, especially in times of a pandemic and increasing mental health burden, it seems important to identify residents' MHL deficits. Based on these findings, they should be supported in their access, understanding, assessing and applying of information on mental health as well as their resilience to stress and other mental health issues and the promotion of their mental well-being. To capture MHL in a more comprehensive manner, the HLS-EU-Q47 could be extended by considering recent MHL constructs and definitions, and including all domains (finding, understanding, appraising, and applying information on mental health) and dimensions (management of mental disorders, prevention thereof, and promotion of mental health).

## Data Availability Statement

The datasets for this study are the property of the Careum Foundation and the Department of Health of the Canton of Zurich and can be received on reasoned request.

## Ethics Statement

Ethical review and approval was not required for the study on human participants in accordance with the local legislation and institutional requirements. Written informed consent for participation was not required for this study in accordance with the national legislation and the institutional requirements.

## Author Contributions

MS, SD, and DN-F conceptualized this study, developed the research question, and were responsible for project administration. MS performed the data analysis and literature research. MS, SD, and RJ prepared the original draft of the article. DN provided critical review of the concept and the manuscript. All authors revised it critically, approved the final manuscript and agreed to be personally accountable for their own contribution to the article and to ensure the accuracy or integrity of any part of the work.

## Funding

Data was collected in the course of the Health Literacy Survey Zurich 2018 which was realized by the Careum Foundation and the Department of Health of the Canton of Zurich.

## Conflict of Interest

The authors declare that the research was conducted in the absence of any commercial or financial relationships that could be construed as a potential conflict of interest.

## Publisher's Note

All claims expressed in this article are solely those of the authors and do not necessarily represent those of their affiliated organizations, or those of the publisher, the editors and the reviewers. Any product that may be evaluated in this article, or claim that may be made by its manufacturer, is not guaranteed or endorsed by the publisher.

## Abbreviations

HLS-CH-15: Swiss Health Literacy Survey
HLS-ZH-18: Health Literacy Survey Zurich.
